# Whole Genome Sequencing Unravels New Genetic Determinants of Early-Onset Familial Osteoporosis and Low BMD in Malta

**DOI:** 10.3390/genes13020204

**Published:** 2022-01-23

**Authors:** Chanelle Cilia, Donald Friggieri, Josanne Vassallo, Angela Xuereb-Anastasi, Melissa Marie Formosa

**Affiliations:** 1 Department of Applied Biomedical Science, Faculty of Health Sciences, University of Malta, MSD 2080 Msida, Malta; chanelle.cilia@um.edu.mt (C.C.); angela.a.xuereb@um.edu.mt (A.X.-A.); 2 Centre for Molecular Medicine and Biobanking, University of Malta, MSD 2080 Msida, Malta; donald.friggieri@um.edu.mt (D.F.); josanne.vassallo@um.edu.mt (J.V.); 3 Department of Medicine, Faculty of Medicine and Surgery, University of Malta, MSD 2080 Msida, Malta; 4 Division of Endocrinology, Mater Dei Hospital, MSD 2090 Msida, Malta

**Keywords:** familial osteoporosis, BMD, whole genome sequencing, *SELP*, *TGF-β2*, *ADAMTS20*

## Abstract

Background: Osteoporosis is a skeletal disease with a strong genetic background. The study aimed to identify the genetic determinants of early-onset familial osteoporosis and low bone mineral density (BMD) in a two-generation Maltese family. Methods: Fifteen relatives aged between 28–74 years were recruited. Whole genome sequencing was conducted on 12 relatives and shortlisted variants were genotyped in the Malta Osteoporotic Fracture Study (MOFS) for replication. Results: Sequential variant filtering following a dominant inheritance pattern identified rare missense variants within *SELP, TGF-β2* and *ADAMTS20*, all of which were predicted to be likely pathogenic and participate in osteoimmunology. *TGF-β2* c.1136C>T was identified in five individuals from the MOFS in heterozygosity, four of whom had osteopenia/osteoporosis at the lumbar spine and hip, and/or had sustained a low-trauma fracture. Heterozygosity for the *ADAMTS20* c.4090A>T was accompanied by lower total hip BMD (*p =* 0.018) and lower total serum calcium levels in MOFS (*p* < 0.01), recapitulating the findings from the family. Women carrying at least one copy of the alternative allele (TC/CC) for *SELP* c.2177T>C exhibited a tendency for lower lumbar spine BMD and/or wrist fracture history relative to women with TT genotype. Conclusions: Our findings suggest that the identified variants, alone or in combination, could be causal factors of familial osteoporosis and low BMD, requiring replication in larger collections.

## 1. Introduction

Osteoporosis is a complex, metabolic skeletal disease characterised by a reduction in bone mass and impaired bone microarchitecture resulting in increased bone fragility [1,2]. Bone mineral density (BMD) measurement by dual-energy X-ray absorptiometry (DXA) remains the accepted gold-standard for the non-invasive diagnosis of osteoporosis [3,4]. Fractures are the major clinical consequence of osteoporosis, with those of the hip, vertebrae, humerus and wrist being the most common, debilitating and costly. Worldwide, osteoporosis is estimated to affect around 200 million people aged 50 years and older [5], and is brought on by an increased rate of bone resorption superseding bone formation, age-derived upsurge of osteoblast and osteocyte apoptosis or the inability to achieve peak bone mass during growth [6]. The underlying pathogenesis is influenced by a multitude of factors ranging from lifestyle factors (such as diet and physical activity), medication and co-existing diseases, and several genetic determinants identified through family- and population-based studies [7,8,9,10,11]. Advancements in sequencing technologies accompanied by the availability of high-throughput sequencing have further enabled the identification of genetic factors that have, in turn, widened the knowledge on the complexity of bone biology and its governing factors. Yet, to date, these findings are only able to explain around 5% of the genetic variance [10], prompting the need for more studies.

Twin and family studies have been crucial in unravelling genes responsible for large effects, some of which have been successfully replicated at the population level with variable BMD phenotypes. An example of such a gene is *LRP5*, primarily identified as the causal defective gene in the development of Idiopathic Juvenile Osteoporosis [12] and Osteoporosis-Pseudoglioma syndrome (OPPG) [13]. Two widely studied *LRP5* missense variants (p.Val667Met; rs4988321 and p.Ala1330Val; rs3736228) have reportedly been associated with lumbar spine (LS) and hip osteoporosis across different ethnic populations [14,15,16,17,18]. Nonetheless, other identified genes with a clear-cut role in bone biology remain family-specific, perhaps requiring large collaborative efforts to increase population numbers and achieve replication. *WNT1,* involved in osteogenic differentiation and bone mineralisation, was detected in families with early-onset osteoporosis and Osteogenesis Imperfecta [19], whereas *PLS3* identified in five Dutch families with X-linked Osteoporosis and fractures is required for the proper formation of filamentous actin bundles [20]. Twin and family studies have confirmed that BMD heritability ranges from 50 to 85% [1,2,21], whilst heritability of fractures ranges from 25 to 68% [22].

In this study, a two-generation Maltese family of 15 individuals having three relatives with osteoporosis was recruited. Whole genome sequencing (WGS) was performed on 12 selected relatives, with the generated data subjected to sequential filtering steps identifying three rare missense variants in *SELP, TGF-β2,* and *ADAMTS20* as potentially damaging and segregating alone or in combination in relatives with osteoporosis and low BMD. The gene variants were genotyped in a case-control collection using polymerase chain reaction (PCR) followed by restriction fragment length polymorphism (RFLP) and real-time PCR with the aim of replicating the findings at the population level. The scope of this study was to enable a better insight into the genetic architecture of osteoporosis and low bone mass that could possibly help identify new potential drug targets.

## 2. Materials and Methods

### 2.1. Ethical Compliance

This study was approved by the University Research Ethics Committee of the University of Malta (UREC No. 18/2010) and written informed consent was obtained from every participating research subject following good ethical guidelines.

### 2.2. Recruitment of the Maltese Pedigree

**Figure 1:**
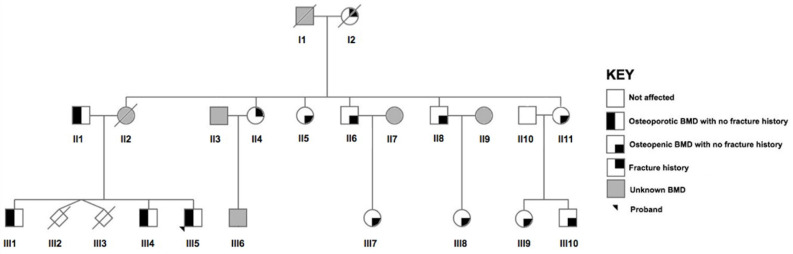
Pedigree of the two-generation Maltese family. Roman numerals indicate generation, whereas Arabic numbers indicate the order. The arrow indicates the proband. Squares represent male relatives, females are depicted in circles, whereas diamonds signify a stillbirth with an unknown gender. Gestational age of the latter is unknown. A diagonal arrow indicates that the individual is deceased and no biological material was available for analyses. A half black symbol represents the presence of osteoporosis, whereas a quarter shading represents osteopenia or a low-trauma fracture history. Relatives with no bone mineral density (BMD) measurements are shaded in grey and marked as ‘unknown’.

A two-generation family of 15 relatives with ages ranging from 28 to 74 years was recruited. Clinical characteristics including BMD measurements and fracture history are shown in Table 1, whereas an overview of the pedigree is displayed in Figure 1. The proband (III5) was a male diagnosed with osteoporosis at the LS and femoral neck, FN at the age of 32. He was referred for a BMD scan following a few years of dull, continuous pain in his lower back, but no vertebral fractures were observed from radiographic imaging. Following his diagnosis, the proband’s older brothers were also subsequently found to be osteoporotic at the ages of 36 years (III4) and 47 years (III1). Their deceased mother (II2) had suffered complete edentulism (loss of teeth) at the young age of 30 years. Unfortunately, no BMD measurements or DNA were available at the time of the study. Her mother (I2) had reportedly sustained a low-trauma hip fracture in her 50s and died as a result of fracture complications. No DNA was available for testing. At the age of 74 years, the proband’s father (II1) was found to have osteoporosis at the LS, which could have been brought on or complicated by several years of undiagnosed and untreated type II diabetes mellitus. He stated that he had no family history of osteoporosis but a positive family history of diabetes. Relative III10 had an elevated oestrogen level and an obese body mass index (BMI = 54.8 kg/m^2^). Relative II4 had sustained a low-trauma wrist fracture at the age of 70. However, no BMD measurements were available for this relative, her son (III6) or spouse (II3). Moreover, no DNA could be collected from relative II3. None of the of relatives had extraskeletal abnormalities or osteogenesis imperfecta.

### 2.3. BMD Measurements and Phenotype Definition

BMD measurements by DXA of the post-anterior LS (L2-L4) and left hip (including FN and total hip, TH) were performed on 13 of the 15 recruited relatives using the Hologic Horizon-Wi densitometer (Hologic Incorporation, Marlborough, MA, USA) situated at the Bone Density Unit within Malta’s main general hospital. The World Health Organisation (WHO) diagnostic criteria were used to define osteoporosis [3] based on the T-scores and Z-scores derived from BMD measurements. Osteoporosis was defined using a T-score below or equal to −2.5 standard deviations (SD; representing 2.5 SD below the young adult mean) at the LS and/or FN in postmenopausal women and men aged over 50 years, or a Z-score of −2.0 SD or less (age, gender and ethnicity matched) at the LS and/or FN in pre-menopausal women and all members under the age of 50 years [19]. Osteopenia was defined as a T-score of -1.0 to −2.4 SD, whereas a T-score of −1.0 SD or more implied a normal BMD [3]. The proband had an LS T-score of −3.7 and Z-score of −3.6, and an FN T-score of −2.6 and Z-score of −2.3.

### 2.4. Collection of Anthropometric Data and Biological Samples

Demographic and lifestyle factors, including age at recruitment, age at menopause (for postmenopausal women), dietary habits (such as calcium and vitamin D, C and K intake, and milk consumption), smoking history and daily alcohol intake, as well as a thorough medical history, including medication use and history of low-trauma fractures, were obtained through an interview-led questionnaire and review of their medical records. BMI was calculated using a combined calibrated stadiometer and balance scales (to the nearest 0.05 kg; AE Adam^®^ MDW 300L, Oxford, CT, USA) to measure height (in centimetres) and weight (in kilograms), respectively. Classification of BMI was based on the ‘WHO Global Database on BMI’ [23,24]. Where possible, blood and urine samples were collected for biochemical investigations to exclude the presence of secondary osteoporosis. This included a complete blood screen, erythrocyte sedimentation rate, renal profile (assessing the levels of urea, creatinine and electrolytes), liver profile (total protein, albumin and liver enzymes), lipid profile (total cholesterol, triglycerides, high-density lipoprotein and low-density lipoprotein), hormone profile (consisting of free thyroxine, thyroid stimulating hormone, luteinising hormone, follicle stimulating hormone, oestradiol, testosterone, cortisol and prolactin), total serum calcium, ionised calcium, phosphate, 25-hydroxy vitamin D, parathyroid hormone (PTH), haemoglobin A1c, random blood glucose and a coeliac screen. The tests were performed at the Pathology Department within Malta’s main general hospital. Whole blood in ethylenediaminetetraacetic acid (EDTA) was also collected for DNA extraction. In the case of relatives residing outside of Malta (II4 and III6), saliva samples were collected using the Oragene OG-500 saliva kits (DNA Genotek, Ontario, Canada) for DNA extraction.

### 2.5. DNA Extraction and WGS

Genomic DNA was extracted from peripheral white blood cells using a modified protocol of the salting-out technique [25], and from saliva using an alcohol precipitation method following the Oragene protocol (DNA Genotek). The quality and quantity were assessed using the Nanodrop 2000c UV Spectrophotometer (Thermo Fisher Scientific, Waltham, MA, USA) and the Qubit 3.0 fluorometer (Thermo Fisher Scientific, Waltham, MA, USA). The 12 most genetically informative relatives from the pedigree were selected for WGS, which also included an unaffected healthy control to aid in gene variant filtering. WGS was carried out on the BGISEQ-500 sequencing platform at the Beijing Genomics Institute (BGI) Tech Solutions Co. Limited, Hong Kong, China using the DNA Nanoballs technology for the generation of the library and the combined primer anchor synthesis for sequencing of nucleotides.

### 2.6. Bioinformatics Processing and Data Analysis

A bioinformatics pipeline was applied to call and annotate single nucleotide variants (SNVs), and insertions and deletions (InDels). In brief, raw reads in the form of FASTq files were aligned to the human reference genome Build GRCh37/UCSC hg19 using Burrows-Wheeler Aligner v0.7.12 [26]. The generated binary alignment map (BAM) files were run through Picard tools v1.40 to sort, merge and index the generated reads, and remove any duplicated reads. The Gene Analysis Toolkit (GATK) v3.3.0 [27] was used to call SNVs and InDels separately, and to generate base-quality score recalibration. An average Q30 score of 89.61% and a 20X coverage of 99.49% were obtained. The called variants were annotated using ANNOVAR [28] providing: (i) an alternative allele frequency from four public databases, including the Single Nucleotide Polymorphism Database (dbSNP) Build 141 [29,30], 1000 Genomes Project [31], GO Exome Sequencing Project of the Heart, Lung and Blood Institute 6500 [32] and Genome Aggregated Database (gnomAD, vr2.1) [33]; (ii) conservation scores according to the Genomic Evolutionary Rate Profiling (GERP) score, which generates estimates of evolutionary conservation of the variants across different species using a maximum likelihood evolutionary rate estimation [34]; (iii) in silico prediction of amino acid changes giving an indication of deleteriousness according to scale-invariant feature transform (SIFT, v5.2.2, [35]), Polymorphism Phenotyping v2 (Polyphen2, v2.2.2, [36,37]), MutationTaster [38], Mutation Assessor [39] and Variant Effect Scoring Tool v3.0 (VEST3, [40]). Other in silico prediction tools provided pathogenicity scores for all SNVs and InDels, including Combined Annotation Dependent Depletion (CADD, [41]), MetaLR and MetaSVM [42] and Protein Variation Effect Analyzer (PROVEAN) v1.1.3 [43]. Variants deposited in the ClinVar database [44] were also flagged together with any reported clinical significance.

Individual variant call format (VCF) files were generated for each sequenced relative for both SNVs and InDels. The individual VCF files of the coding SNVs and InDels were merged into one file using Python (https://github.com/df-80/ngs_merge, created on 6 February 2018; accessed on 20 October 2020) for gene variant filtering. Although the main focus of the study was to identify variants residing in protein coding regions, more commonly referred to as the ‘low hanging fruit’, WGS was preferred over whole exome sequencing (WES). WGS offers a better allele ratio compared to WES, has a better detection rate of InDels and structural variants, and does not require a capture method that can inadvertently introduce bias. Furthermore, if nothing of significance is detected in the coding regions, the non-coding areas can be further scrutinised [45,46].

Following this approach, WGS yielded a total of 42,854 SNVs and 1957 InDels. Sequential variant filtering included: (i) dominant inheritance in confirmed affected (osteoporotic) relatives (III1, III4 and III5) retaining heterozygous variants, and removal of all variants fitting a dominant or recessive inheritance pattern for the unaffected relative (II10); (ii) removal of benign SNVs and InDels keeping missense, splice acceptor and splice donor (within 10 nucleotides into the intron-exon junction), start loss, stop gain and stop loss SNVs, as well as frameshift variants (including those in splicing regions or resulting in start/stop loss) and inframe InDels; (iii) retaining of gene variants with an observed alternative allele frequency of ≤1% in all population-based allele frequency databases assuming the presence of rare and possibly family-specific variants; (iv) retaining of missense variants predicted to be deleterious by most in silico tools, having a CADD score of >15, predicted to be likely pathogenic or pathogenic by the American College of Medical Genetics and Genomics/Association for Molecular Pathology (ACMG/AMP scores of 4 and 5) [47]), and being evolutionary conserved; (v) removal of documented false-positive variants (known as frequent hitters) [48,49]; and (vi) retaining of variants falling in genes with a plausible role in bone biology or expressed in bone tissue according to the Mouse Genome Informatics, MGI ([50,51], http://www.informatics.jax.org, accessed on 9 April 2021), International Mouse Phenotyping Consortium, IMPC ([52], https://www.mousephenotype.org/ accessed on 9 April 2021) and Musculoskeletal Knowledge Portal ([53], https://msk.hugeamp.org/ accessed on 9 April 2021). The shortlisted variants were genotyped by bidirectional Sanger sequencing (LGC Genomics, Berlin, Germany) in all the recruited relatives using the BioDye Terminatory method (Applied Biosystems, Waltham, CA, USA) and the results were analysed with Codon Code Aligner software v9.0 (CodonCode Corporation, Centerville, MA, USA) to determine their segregation across the family.

### 2.7. Genotyping of Shortlisted Variants in a Maltese Population Study

A total of three shortlisted SNVs were genotyped in the Malta Osteoporotic Fracture Study (MOFS), a case-control collection of 1045 Maltese postmenopausal women (two-generation Maltese) with ages ranging from 41 and 79 years matched in 10-year age groups. Cases were women with a low-trauma fracture history (*n* = 268, 26%), whereas controls were women who did not sustain a fracture further subdivided into three groups according to their BMD T-score measurements (normal BMD at all measured anatomical sites [*n* = 229, 22%], osteopenic BMD at the LS and/or FN [*n* = 266, 25%] and osteoporotic BMD at the LS and/or FN [*n* = 282, 27%]). The study, described in more detail elsewhere [54], aimed to investigate the biochemical, clinical, lifestyle and genetic factors contributing to osteoporosis and fragility fractures in Malta. Two variants were genotyped by allelic discrimination real-time PCR; *ADAMTS20* c.4090A>T (rs138035327) was genotyped by LGC Genomics using Competitive Allele Specific PCR (KASP^TM^) with genotyping data graphically viewed by SNPviewer2 v3.2.2.16 (LGC Genomics), whereas *SELP* c.2177T>C (rs754086574) was genotyped in-house on the Bio-Rad CFX96 Real-Time PCR Detection System (Bio-Rad, Hercules, CA, USA) using a predesigned TaqMan genotyping assay (Applied Biosystems, Waltham, CA, USA; ID:C_362473276_10) according to the manufacturer’s instructions. PCR followed by RFLP was used to genotype the *TGF-β2* c.1136C>T (rs773943514) variant. Primer sequences were designed *de novo* (Forward primer 5′-3′: TCTGTGCTGGAGCATGCC, Reverse primer 5′-3′: CTGGGCAGGTGAGACTTATGG) with an annealing temperature of 54 °C amplifying a 338 base pair PCR product. The *Hae*III restriction enzyme (New England BioLabs, Ipswich, MA, USA) was used to cut in the presence of the reference C allele with digested fragments resolved on a 2% agarose gel.

### 2.8. Statistical Analysis

Statistical data analysis was performed using the Statistical Package for Social Sciences (SPSS) software v22 (SPSS, Chicago, IL, USA). Genotype and allele frequencies were determined across the entire MOFS collection, as well as in the different sub-groups, with the Hardy Weinberg Equilibrium (HWE) computed in the control group with a normal BMD (*n* = 229) using the Fisher’s Exact Test. Genotype-phenotype distributions for bone-related phenotypic measurements, including BMD levels at the LS, FN and total hip (TH), and biochemical parameters (serum calcium, total serum alkaline phosphatase [ALP] and serum albumin) were determined for the three gene variants, with data presented as medians accompanied by interquartile ranges. Genotype-phenotype distributions are shown for controls with a normal BMD, women with osteopenia and osteoporosis at any measured anatomical sites but without a fracture history, since it might further decrease their BMD [55], fracture cases and the whole MOFS collection. The association of the genotype with individual bone phenotypes was investigated using the Mann–Whitney U test (for non-parametrically distributed data) in controls with a normal BMD, since a low BMD and a fracture history can introduce bias. A *p*-value of less than 0.05 was considered significant. Logistic regression using odds ratios (OR) with 95% CI adjusted confounders were computed to determine the effect of the genotyped variants on BMD at the LS, FN and TH, all-type of low-trauma fracture risk, site-specific fractures and biochemical parameters (divided into tertile intervals based on the distribution in controls with a normal BMD).

## 3. Results

### 3.1. WGS Data Filtering

Stepwise variant filtering following an autosomal dominant inheritance pattern identified three conserved missense SNVs (Table 2) present in heterozygosity in the three affected osteoporotic male siblings (III1, III4 and III5), which were absent in the unaffected control (II10). No InDels were identified following sequential variant filtering.

### 3.2. Description of the Identified Gene Variants

A summary of these three shortlisted rare missense variants can be seen in Table 3, all of which were present in population-based allele frequency databases, having an rs number, and detected at a frequency of <0.01% according to gnomAD. Two gene variants resided on chromosome 1: the (NM_003005.3): c.2177T>C: p.Tyr726Cys (Chr1[GRCh37/hg19]: g.169564040T>C) within exon 13 (of 17) of *SELP* and (NM_001135599.2): c.1136C>T: p.Pro379Leu (Chr1[GRCh37/hg19]: g.218610804C>T) in exon 6 (of 7) of *TGF-β2*. The *TGF-β2* variant has been lifted into (NM_003283.3): c.1052C>T: p.Pro351Leu (Ch1[GRCh38/hg38]: g.218437462). Both variants are predicted to be deleterious or damaging by all in silico tools, and by the ACMG/AMP Guidelines. The *SELP* c.2177T>C variant affects the 726 amino acid residue and resides in a zinc-binding site, whereas *TGF-β2* c.1136C>T alters a cysteine knot. The third variant, *ADAMTS20* (NM_025003.3): c.4090A>T: p.Tyr1364Asn (Chr12[GRCh37/hg19]: g.43821128A>T), is located in exon 27 (from a total of 39), and is predicted to be deleterious by most in silico tools. Evolutionary conservation shows that the variant occurs at a highly conserved position within a β-sheet of the protein structure.

**Figure 2:**
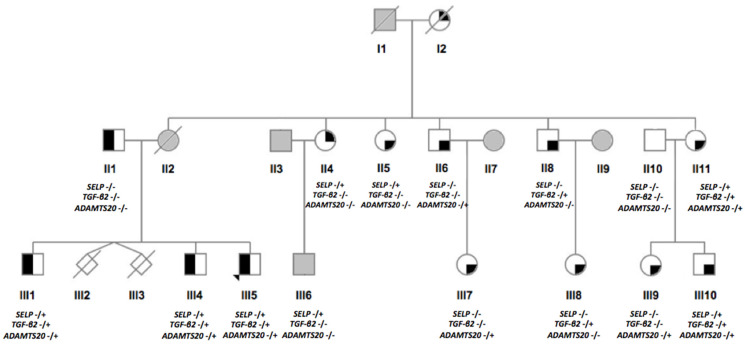
Segregation of the three risk variants confirmed by Sanger sequencing in recruited relatives. The gene name represents the respective gene variants (i.e., *SELP* c.2177T>C, *TGF-β2* c.1136C>T and *ADAMTS20* c.4090A>T). The gene name (in italics) is followed by the genotype, with ‘-/-‘ representing a homozygous reference genotype, whereas ‘-/+‘ represents a heterozygous genotype for each respective variant.

**Figure 3:**
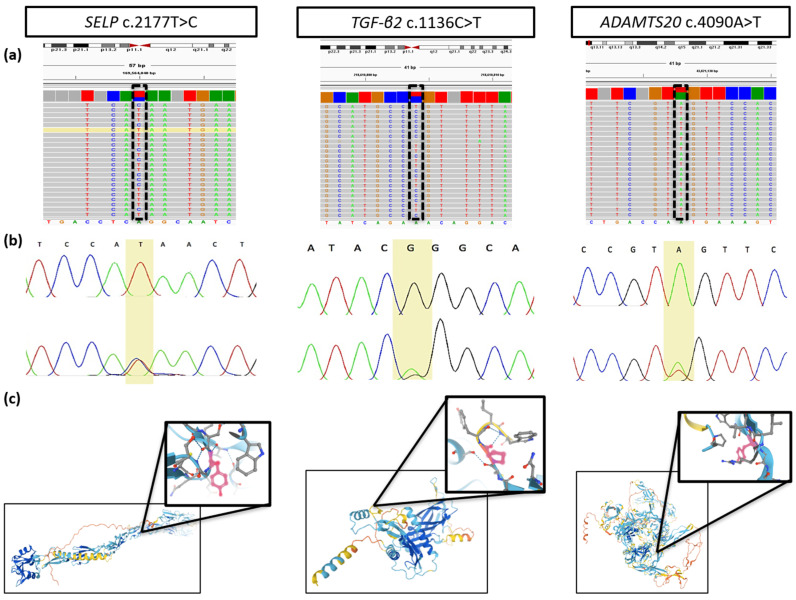
Validation of the shortlisted gene variants. (**a**) Excerpt of the BAM file uploaded in IGV showing the heterozygous change for all three variants, (**b**) Sanger sequencing traces showing a confirmed homozygous reference (top) and heterozygous (bottom) sequence for each of the three risk variants, (**c**) protein structure of P-selectin, TGF-β2 and ADAMTS20 with a focus on the position of the variant within the protein according to AlphaFold Protein Structure Database [57,58].

Segregation of the gene variants in the family was confirmed by Sanger sequencing, few of whom were found carrying one or more of the variants in heterozygosity (Figure 2). The gene variants were also examined in the Integrative Genomics Viewer (IGV, [56]) to confirm the presence of the variant, determine the overall coverage of the selected regions and exclude the possibility of misalignments or inaccurate bioinformatics processing resulting in false-positive hits. The coverage of the sequencing data was consistently above 30X. An excerpt from IGV showing the three variants in heterozygosity is seen in Figure 3a, whereas Sanger sequencing traces showing a normal (top) and mutated (bottom; heterozygous genotype) sequence are displayed in Figure 3b. The affected amino acid in the protein structure as predicted by AlphaFold Protein Structure Database ([57,58], https://alphafold.ebi.ac.uk accessed on 10 November 2021) can be seen in Figure 3c.

### 3.3. Genotyping Results of the Epidemiological Study

Genotype and allele frequencies for the three genotyped variants in the entire MOFS collection and sub-groups can be found in Table 4. Genotyping of the *SELP* c.2177T>C variant in the MOFS collection identified 1028 (98.4%) women with the homozygous reference genotype, 11 (1.1%) carrying the variant in heterozygosity, and 6 (0.6%) with the homozygous alternative genotype. The alternative C allele was present at a frequency of 0.7% in controls, with the highest alternative allele frequency detected in women with an osteopenic BMD (2.1%) followed by women with an all-type of low-trauma fracture history (1.1%). The *TGF-β2* c.1136C>T variant was successfully genotyped in the entire collection. Only five women carrying the variant in heterozygosity were detected, equating an alternative allele frequency of 0.2%, the majority of whom had osteopenia and/or osteoporosis and a non-vertebral fracture history (wrist, humerus and hip). *ADAMTS20* c.4090A>T was also only found present in heterozygosity albeit at a higher alternative allele frequency (T = 3%) than that reported in gnomAD, possibly indicating the presence of a founder effect in the Maltese population. The variant was successfully genotyped in 1012 individuals from the MOFS collection. Women with an osteoporotic BMD but without a fracture history had the highest alternative allele frequency (T = 3.3%) for the *ADAMTS20* c.4090A>T variant. All variants were in HWE.

Phenotype levels according to the different genotypes are displayed in Table 5. In view of the low alternative allele frequencies detected, women with an osteopenic and osteoporotic BMD at any measured site were grouped into one category (labelled as Osteopenia & Osteoporosis). Furthermore, women carrying the heterozygous or homozygous alternative genotype for *SELP* c.2177T>C were combined to increase the number of individuals with the risk allele in the category. Genotype-phenotype associations were computed in controls with a normal BMD to avoid bias. This was only possible for *ADAMTS20* c.4090A>T, with the low sample sizes making the data analysis unfeasible for *SELP* c.2177T>C and *TGF-β2* c.1136C>T. Heterozygosity for the *ADAMTS20* c.4090A>T variant was associated with a lower TH BMD relative to homozygosity for the reference A allele (*p =* 0.018). This was the only significant association detected. Nonetheless, other consistent trends were observed, including a lower serum calcium in women with AT genotype for the *ADAMTS20* c.4090A>T relative to women with the AA genotype. Fracture cases carrying at least one copy of the alternative allele for *SELP* c.2177T>C and *TGF-β2* c.1136C>T had a lower TH BMD.

The effect of the *SELP* and *ADAMTS20* gene variants on BMD (LS, FN and TH) and fracture risk (all-type and wrist) was further assessed using logistic regression analysis. Crude and adjusted ORs with 95% CI together with *p*-values are shown in Table 6. None of the associations reached statistical significance which could be explained by the low sample sizes when analysing rare or low frequency variants.

Trends observed in relation to the total serum calcium (*ADAMTS20* c.4090A>T) were further analysed using logistic regression analysis in the entire MOFS collection. Genotyped variants were analysed in relation to biochemical parameters divided into tertile intervals. Heterozygosity of the *ADAMTS20* c.4090A>T variant was significantly associated with a 2.3-fold and 3-fold increased risk of low and moderate serum calcium levels, respectively, relative to homozygosity for the reference A allele (Table 7).

## 4. Discussion

In the present report, WGS of a two-generation Maltese pedigree with early-onset familial osteoporosis and low BMD identified three rare and evolutionary conserved missense variants within *SELP* (c.2177T>C), *TGF-β2* (c.1136C>T) and *ADAMTS20* (c.4090A>T). The gene variants were subsequently tested in a Maltese case-control collection to determine causality with bone-related phenotypes at the population level. The three variants were predicted to have a deleterious effect on protein function based on multiple independent algorithms or classification guidelines. No widescale population-based genetic association studies have been reported to date in relation to these gene variants and none of the variants were found to be in linkage disequilibrium with other known variants or each other, as is mostly expected for rare variants.

The *SELP* c.2177T>C variant was present in heterozygosity in the three osteoporotic male siblings (III1, III4, III5), as well as relatives II4, II5, II11 and III10, all of whom had mild osteopenia at the LS or a history of a low-trauma wrist fracture (in the case of II4). The variant was also present in III6 whose BMD is unknown. Genotyping of the variants in the MOFS collection identified six unrelated postmenopausal with the homozygous alternative genotype; four had osteopenia at the LS and FN (average LS T-score: −1.40, FN T-score: −2.00), whereas two has osteoporosis at the LS (T-score: −2.7), one of whom had sustained a wrist fracture similar to that reported in the family. Moreover, eight women with a low BMD at the LS (average T-score: −1.71) and FN (average T-score: −2.37) and/or a hip fracture history carried the variant in heterozygosity. The variant was also detected in 1.3% (*n* = 3) of women with a normal BMD. However, given their younger age (<55 years), they might develop osteoporosis at a later stage.

The *SELP* gene encodes the P-selectin protein, a 140 kilodalton (kDa) protein, stored in the α granules of platelets, which acts as a cell adhesion molecule on the surfaces of activated endothelial cells that line blood vessels [59]. Selectins, particularly E-selectin, are involved in an ongoing project aimed at producing novel anabolic bone treatment using exofucosylated mesenchymal stem cells to increase their differentiation into osteoblasts promoting bone formation [60]. The gene variant identified in the study, results in the amino acid substitution of a tyrosine (an essential amino acid with a hydrophobic side chain) with cysteine (a non-essential amino acid that forms disulphide bridges to stabilise the protein) at position 726 located in the evolutionary conserved extracellular sushi domain of P-selectin. This evolutionary conserved extracellular domain is characterised by a β-sandwich arrangement [61]; a missense variant in this domain will most likely cause deficiency of one or more of the β-strands, resulting in abnormal protein function. *Selp* knockout mice exhibit an increased susceptibility of collagen-induced arthritis [62,63]. Selectins trigger the initial migratory phase of leucocytes from the vasculature into tissue during the inflammatory process [59,64,65,66,67], highlighting the involvement of *SELP* in inflammation.

The second missense variant identified in heterozygosity in the three osteoporotic relatives resided in *TGF-β2*. Additionally, four relatives (II8, II11, III8 and III10) with an osteopenic LS and/or FN BMD were found carrying the heterozygous genotype for this same variant. The variant, although linked to Holt-Oram syndrome (OMIM 142900) in ClinVar [44], does not have any supporting functional evidence to validate the predicted disruptive effect on the protein structure and function, and is, thus, labelled as a variant of ‘uncertain significance’. Furthermore, there are no reports of individuals with TGF-β2 disease harbouring the gene variant. Yet, its association with Holt-Oram syndrome, a rare autosomal disease characterised by skeletal and heart defects [68], suggests a potential role in bone pathophysiology. In the MOFS collection, five postmenopausal women with the *TGF-β2* c.1136C>T heterozygous genotype were detected. Out of the five identified women, three had a history of at least one low-trauma non-vertebral fracture (wrist, humerus and/or hip fracture) accompanied by an osteopenic/osteoporotic BMD (average LS T-Score: −2.4, FN T-score: −2.8, TH T-score: −2.8), a 69-year old female had osteopenia at LS (T-score: −2.4) and hip (FN T-score: −1.1, TH T-score: −1.1), whereas the fifth individual, a 56-year old female, had a normal BMD at all anatomical sites (LS T-score: −0.3, FN T-score: 0.5, TH T-score: 1.5). The variable phenotypes seen in the relatives and unrelated women could possibly be explained by incomplete penetrance, development of the disease at a later stage, differing temporal and spatial activation of *TGF-β2*, or presence of risk factors, modifier genes and gene variants that might be altering the presenting phenotype [69,70,71].

The TGF-β signalling pathway plays a critical role in the control and development of bone and cartilage, modulating cell proliferation, differentiation, apoptosis, adhesion and migration, as well as enhancing bone extracellular matrix proteins, ECMs [72,73,74,75]. The multifunctional cytokine can also regulate other signalling pathways (e.g., Wnt and Notch) and factors (e.g., RUNX2 and FGFs) to influence osteoblast/osteoclast balance and pathogenesis [72]. The TGF-β family consists of three closely related growth factors (TGF-β1–3) that activate or inhibit cell proliferation and growth depending on context and concentration. *Tgf-β2* knockout mice exhibit abnormal skeletogenesis (in both cranial and non-cranial sites of the body), with a 66% chance of death before or during birth as a result of accumulated developmental defects [76].

TGF-β2 controls the formation of blood vessels and regulates muscle tissue, wound healing, body fat development and immune system function [77]. A 4.7Mb deletion in the *TGF-β2* has reportedly been associated with familial osteoporosis and features of Loeys-Dietz syndrome type IV (OMIM 613795), with affected individuals developing fractures of the pelvis and ribs in adulthood [78]. The 47.7 kDa TGF-β2 ligand contains a cysteine knot structural motif consisting of an embedded ring formed by two disulphide bonds and their connecting backbone segments that are threaded by a third disulphide bond. This motif, together with the β-sheet structure, appears to function in quaternary protein stabilisation [79]. Consequently, the physiochemical change brought on by the variant identified in this study, which is a proline (with its unique cyclic ring giving it conformational rigidity) to a leucine amino acid at position 379, results in the disruption of the cysteine knot structure, which is in turn hypothesised to alter TGF-β2 function.

The heterozygous missense variant *ADAMTS20* c.4090A>T was identified in eight relatives from the investigated pedigree (II6, II11, III1, III4, III5, III7, III9 and III10), all having a low LS and/or FN BMD according to their T-scores and Z-scores. According to gnomAD, the variant has an alternative allele frequency of around 0.08% in the European population, which is much lower than that detected in MOFS (T = 3%), possibly suggesting the presence of a founder effect. This can be attributed to the admixture of Maltese inhabitants with other populations from Mediterranean and Northern European regions that colonised Malta in the past, as well as the recent admixture due to migrations from Africa and Asia, which has subsequently overcome the geographical barriers [80,81,82]. Other studies have supported the hypothesis of a founder effect in the Maltese population [83,84,85,86].

In the MOFS collection, heterozygosity for the *ADAMTS20* c.4090A>T variant was significantly associated with a lower TH BMD (Table 5; *p =* 0.018), as well as a 2.3-fold (*p =* 0.032) and 3-fold (*p* = 0.004) increased risk of low and moderate serum calcium levels, respectively (Table 7) relative to women with the homozygous reference genotype. Relatives carrying the variant in heterozygosity also had lower serum calcium levels (mean serum calcium = 2.31 mmol/L [±0.08]) compared to those with the homozygous reference genotype, recapitulating these findings. PTH levels were within the normal reference range for all relatives.

ADAMTS family proteins are multidomain extracellular protease enzymes that include 19 members subdivided on the basis of their known substrates. The proteases control the structural properties of the ECM, organogenesis, tissue organisation and cell signalling [87]. They have also been implicated in the processing of procollagens and von Willebrand factor (vWf), cleavage of aggrecan, connective tissue organisation, arthritis, coagulation, inflammation and angiogenesis [87]. Several variants in genes coding for ADAMTS proteins reportedly influence fibrillin microfibrils assembly and abundance that subsequently alter TGF-β mediated signalling [88,89]. ADAMTS20, a 214.7 kDa protein, forms part of the aggrecanase subgroup, as it can cleave extracellular proteins such as aggrecan (encoded by *ACAN*). *ACAN* expression is reportedly upregulated following calcitriol treatment, with proper levels of calcium and PTH required for prolonged biosynthesis of aggrecan [90]. Thus, the association observed between *ADAMTS20* c.4090A>T and levels of serum calcium brings to light a possible interaction that deserves further investigation. Aggrecan, a proteoglycan, plays an essential role in the function of articular cartilage. Agents that prevent aggrecan degradation and restore its production might be critical for the treatment of early-onset osteoarthritis [91]. Mice harbouring two *Adamts20* knockout alleles develop syndactyly, interdigital webbing, white-spotting and defective sphenoid bone closure (the latter observed in combination with *Adamts9* deletion) [50,92,93]. Recently, it has been shown that inactivation of *B3GLCT* affects the secretion of ADAMTS20 in Peters plus syndrome (OMIM 261540). *B3glct* mutant mice exhibited reduced bone growth and thinner growth plates in the long bones, scapula, digits and cranium compared to wild-type littermates, further suggesting a role in bone homeostasis [94].

The *ADAMTS20* c.4090A>T (p.Tyr1364Asn) variant is located in the region that codes for the thrombospondin type-1 repeat (TSR). These repeats are important for secondary protein structure formation, and are considered the ‘trademark’ of thrombospondins [95]. These areas very highly conserved across different species thanks to the process of exon shuffling and are believed to function as a sulphate glycosaminoglycan-binding domain, linking and stabilising proteins, such as aggrecan with hyaluronan expressed in connective tissue [96]. Chromatin Immunoprecipitation Sequencing (ChIP-Seq) data have showed that the *ADAMTS20* c.4090A>T variant affects the binding of Ets transcription factor motifs [97]. The Ets transcription factor family is involved in multiple developmental processes at the cell, tissue and organ level [98]. The products of Ets1 and Ets2 are expressed in developing osteoblasts, where Ets1 is expressed mostly in pre-osteoblasts and Ets2 is expressed at higher levels in post-mitotic mature osteoblasts. This evidence supports the notion that altered Ets transcription factor binding will inadvertently affect osteoblast differentiation and bone development [99].

The three identified missense variants could all be potentially contributing to the underlying pathogenesis observed in this pedigree, either alone or in combination. The presence of a single variant, as opposed to a combination of all three, could explain the mild to moderate phenotypes observed in the relatives, with those carrying all three variants having a more deleterious effect and, hence, osteoporosis [100]. Nonetheless, two relatives (II11 and III10) with a milder phenotype were also found carrying all three variants, possibly due to incomplete penetrance resulting from a combination of genetic, epigenetic, environmental and lifestyle factors [101,102]. Indeed, the elevated oestrogen level and weight (BMI = 54.8 kg/m^2^) of relative III10 could have acted as protective contributors counteracting the combined deleterious effects of the genetic variants and low BMD [103]. Besides incomplete penetrance, the presence of overlapping phenotypes and variable expressivity could have led to individuals with the same genotype expressing different BMD characteristics [104]. Other gene variants residing in the non-coding regions might also explain the osteoporotic BMD in the three male siblings and are yet to be explored.

**Figure 4:**
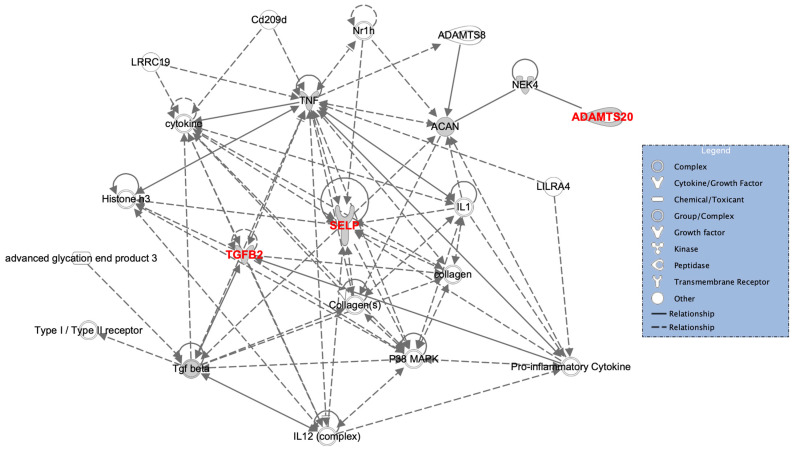
The interaction network highlighting the interaction of P-selectin, TGF-β2 and ADAMTS20 (shown in red) with TNF-α and pro-inflammatory cytokines using Ingenuity Pathway Analysis^®^.

All three encoded proteins, P-selectin, TGF-β2 and ADAMTS20, are directly and indirectly related to one another. As seen in Figure 4, the proteins are linked via TNF-α signalling. SELP and TGF-β2 have a direct link, whilst ADAMTS20 is indirectly related to TNF-α through NEK4 and aggrecan. Studies have shown that P-selectin is negatively correlated with TNF-α in adults, implying that a low P-selectin level (possibly brought on by deleterious protein-impacting variants), would be accompanied by higher TNF-α levels [105]. TNF-α is an adipokine and cytokine, which together with other proinflammatory cytokines, promotes bone catabolism. This constitutes the field of osteoimmunology describing the interactions between the immune system and bone metabolism [106]. Upregulation of TNF-α and interleukins (e.g., IL-1, IL-6, IL-7, IL-8, IL-11, IL-12 and IL-15) induces (i) the inhibition of osteoblastogenesis via p38 MAPK and reduced bone formation with decreased expression of matrix forming proteins such as type I collagen, (ii) the activation of NFkB driving osteoclast differentiation and (iii) the increased resorptive capacity of recruited osteoclasts [107,108,109]. This ties all the shortlisted genes to the concept of osteoimmunology.

A limitation to consider in this study is the limited sample size to achieve replication at the population level. Although the MOFS collection includes over a 1000 research subjects, this was not large enough to statistically determine any significant associations, in particular for the *TGF-β2* c.1136C>T variant. Some of the trends observed could be confirmed if larger collections are used, possibly through participation in a large international consortium. Further studies of the three risk variants are proposed using *in vitro* and *in vivo* models to functionally assess the predicted impact. Assessment will also be extended to include the non-coding part of the genome to get a better understanding of the mechanisms of osteoporosis and low BMD in the investigated pedigree.

## 5. Conclusions

In conclusion, this report has described three rare missense variants in *SELP, TGF-β2* and *ADAMTS20* that alone or in combination could potentially be the underlying genetic determinant(s) contributing to early-onset familial osteoporosis and low BMD in the two-generation Maltese pedigree. The role of the variants in BMD and fracture risk was highlighted at the population level, with associations or trends seen with BMD at different anatomical sites, fracture risk and biochemical parameters. Some of the associations recapitulated the findings observed in the family, including low serum calcium levels in individuals carrying the heterozygous genotype for the *ADAMTS20* c.4090A>T, osteopenia and osteoporosis at the LS, and a wrist fracture history in women carrying at least one copy of the *SELP* c.2177T>C alternative allele, and an overall low (osteopenia/osteoporosis) LS and FN BMD in women with the CT genotype for the *TGF-β2* c.1136C>T variant. Although the sample size did not allow for associations to reach statistical significance, the trends observed provided supporting suggestive evidence for the variants’ role in the genetic architecture of bone disease, possible through participation in osteoimmunology.

## Figures and Tables

**Table 1:** Clinical characteristics of the recruited relatives.

ID	Sex	Age (Years)	BMI (kg/m^2^)	LS T-Score	LS Z-Score	FN T-Score	FN Z-Score	TH T-Score	TH Z-Score	Fracture Sustained	WGS
II1	M	74	27.9	−2.6	−1.6	−1.7	−0.3	−0.7	0.1	-	✓
II4	F	72	-	-	-	-	-	-	-	Wrist at 70 years	✓
II5	F	71	30	−1.3	1.0	−0.6	1.3	0.1	1.6	-	
II7	M	69	24.8	−1.6	−0.7	−1.7	−0.5	−1.4	−0.8	-	✓
II8	M	66	27.9	−1.7	−0.8	−1.8	−0.7	−0.9	−0.3	-	✓
II10	M	69	36.9	1.8	2.7	−0.4	0.7	0.1	0.7	-	✓
II11	F	63	35.6	−1.4	0.3	−0.4	1.1	−0.2	0.9	-	
III1	M	47	21.0	−2.2	−1.8	−2.9	−2.2	−1.9	−1.5	-	✓
III4	M	36	22.5	−2.5	−2.4	−1.5	−0.8	−1.2	−1.0	-	✓
III5	M	32	22.5	−3.7	−3.6	−2.6	−2.3	−1.7	−1.5	-	✓
III6	M	52	-	-	-	-	-	-	-	-	✓
III7	F	42	24.7	−1.6	−1.2	−1.3	−1.0	−1.3	−1.0	-	✓
III8	F	28	18.3	−1.3	−1.3	−1.5	−1.4	−1.4	−1.4	-	✓
III9	F	43	27.9	−0.8	−0.4	−1.7	−0.3	−0.7	0.1	-	✓
III10	M	39	54.8	−1.3	−1.3	0.8	1.2	0.0	0.2	-	

Abbreviations: BMI = Body Mass Index, LS = Lumbar Spine, FN = Femoral Neck, TH = Total Hip, WGS = Whole Genome Sequencing

**Table 2:** Sequential filtering steps applied to the merged vcf files of the SNVs and Indels.

Filtering Step	Single Nucleotide Variants (SNVs)	Insertions & Deletions (InDels)
Total number of variants	42,854	1957
Zygosity filtering following a dominant inheritance pattern for affected relatives (III1, III4, III5); removal of all variants fitting a dominant or recessive inheritance pattern in the unaffected control (II10)	1902	55
Removal of benign variants including synonymous, deep intronic variants	936	18
Retaining of gene variants with an observed alternative allele frequency of ≤1% in population-based allele frequency databases	34	18
Retaining of missense variants predicted to be deleterious by most in silico prediction tools	33	18
Removal of ‘frequent hitters’	32	18
Removal of genes with no association to bone or fractures in literature	3	0

**Table 3:** Summary of the three shortlisted risk variants following WGS.

Gene	Variant (Rs Number)	AAF (GnomAD)	Chromosome: Position ^1^	Coverage	Amino Acid Change	CADD Score	Polyphen-2	SIFT	Mutation Taster	Mutation Assessor	Vest3	Clinvar	ACMG Score
*SELP*	c.2177T>C (rs754086574)	C: 0.0000756	1: 169564040	49	p.Tyr726Cys	23.5	D	D	D	D	D	NR	4 ^2^
*TGF-β2*	c.1136C>T (rs773943154)	T: 0.0000159	1: 218610804	47	p.Pro379Leu	25.0	D	D	D	D	D	US	4
*ADAMTS20*	c.4090A>T (rs138035327)	T: 0.0008091	12: 43821128	46	p.Tyr1364Asn	24.3	D	B	D	D	D	NR	4

^1^ According to GRCh37/hg19. ^2^ Score of 4 labelled as ‘Likely Pathogenic’. Abbreviations: AAF = Alternative Allele Frequency, D = Damaging/Deleterious, B = Benign, NR = Not Reported, US = Uncertain significance.

**Table 4:** Genotype and allele frequencies of the three shortlisted variants in the MOFS collection.

Variant	Genotype			AAF	HWE ^1^
	Homozygous Reference *n* (%)	Heterozygous *n* (%)	Homozygous Alternative *n* (%)		
*SELP* c.2177T>C Controls Normal BMD Osteopenic BMD ^2^ Osteoporotic BMD ^3^ All-type Fractures Whole collection	TT: 226 (98.7) TT: 259 (97.4) TT: 280 (99.3) TT: 263 (98.1) TT: 1028 (98.4)	TC: 3 (1.3) TC: 3 (1.1) TC: 1 (0.4) TC: 4 (1.5) TC: 11 (1.1)	CC: 0 CC: 4 (1.5) CC: 1 (0.4) CC: 1 (0.4) CC: 6 (0.6)	C: 0.007 C: 0.021 C: 0.005 C: 0.011 C: 0.011	*p* = 1.00
*TGF-β2* c.1136C>T Controls Normal BMD Osteopenic BMD ^2^ Osteoporotic BMD ^3^ All-type Fractures Whole collection	CC: 228 (99.6) CC: 265 (99.6) CC: 282 (100) CC: 265 (98.9) CC: 1040 (99.5)	CT: 1 (0.4) CT: 1 (0.4) CT: 0 (0.0) CT: 3 (1.1) CT: 5 (0.5)	TT: 0 TT: 0 TT: 0 TT: 0 TT: 0	T: 0.002 T: 0.002 - T: 0.006 T: 0.002	*p* = 1.00
*ADAMTS20* c.4090A>T Controls Normal BMD Osteopenic BMD ^2^ Osteoporotic BMD ^3^ All-type Fractures Whole collection	AA: 205 (94.0) AA: 247 (95.7) AA: 258 (93.5) AA: 245 (94.2) AA: 955 (94.4)	AT: 13 (6.0) AT: 11 (4.3) AT: 18 (6.5) AT: 15 (5.8) AT: 57 (5.6)	TT: 0 TT: 0 TT: 0 TT: 0 TT: 0	T: 0.030 T: 0.021 T: 0.033 T: 0.029 T: 0.028	*p* = 1.00

^1^ HWE computed in controls with a normal BMD using the Fisher’s exact test. ^2^ Women with an osteopenic BMD without a fracture history. ^3^ Women with an osteoporotic BMD without a fracture history. Abbreviations: AAF = Alternative Allele Frequency, HWE = Hardy Weinberg Equilibrium

**Table 5:** Levels of bone-related phenotypic measurements according to the three genotyped variants in MOFS.

Phenotype (Units)	Normal BMD ^1^		Osteopenia & Osteoporosis ^2^		All-Type Fractures		Whole Collection	
***SELP* c.2177T>C**	**TT** **(*n* = 226)**	**TC/CC** **(** ***n*** **= 3)**	**TT** **(** ***n*** **= 539)**	**TC/CC** **(** ***n*** **= 9)**	**TT** **(** ***n*** **= 263)**	**TC/CC** **(** ***n*** **= 5)**	**TT** **(** ***n*** **= 1028)**	**TC/CC** **(** ***n*** **= 17)**
LS BMD (g/cm^2^)	1.13 (1.05–1.23)	1.36 (1.15–1.46)	0.92 (0.84–0.99)	0.95 (0.86–0.99)	0.91 (0.82–1.01)	0.91 (0.85–1.07)	0.96 (0.86–1.07)	0.99 (0.89–1.07)
FN BMD (g/cm^2^)	0.89 (0.85–0.97)	0.97 (0.89–0.99)	0.74 (0.67–0.80)	0.76 (0.71–0.82)	0.69 (0.63–0.77)	0.67 (0.58–0.70)	0.76 (0.68–0.86)	0.75 (0.70–0.86)
TH BMD (g/cm^2^)	1.00 (0.91–1.06)	0.98 (0.95–1.06)	0.80 (0.73–0.88)	0.84 (0.77–0.89)	0.76 (0.70–0.85)	0.64 (0.59–0.81)	0.83 (0.74–0.92)	0.85 (0.75–0.93)
Calcium (mmol/L^) 3^	2.40 (2.32–2.46)	2.37 (2.35–2.44)	2.40 (2.34–2.48)	2.39 (2.38–2.53)	2.34 (2.26–2.43)	2.25 (2.14–2.41)	2.39 (2.31–2.46)	2.39 (2.33–2.44)
ALP (U/L) ^4^	165 (132–197)	243 (128–288)	157 (126–185)	192 (163–218)	154 (120–183)	231 (94–243)	157 (126–186)	214 (153–234)
Albumin (g/L) ^5^	44.0 (42.2–45.9)	47.1 (40.9–47.7)	43.8 (41.9–45.8)	43.5 (41.6–46.9)	42.2 (39.9–44.6)	43.4 (38.6–44.7)	43.5 (41.6–45.6)	43.4 (41.3–46.6)
***TGF-β2* c.1136C>T**	**CC** **(** ***n*** **= 228)**	**CT** **(** ***n*** **= 1)**	**CC** **(** ***n*** **= 547)**	**CT** **(** ***n*** **= 1)**	**CC** **(** ***n*** **= 265)**	**CT** **(** ***n*** **= 3)**	**CC** **(** ***n*** **= 1040)**	**CT** **(** ***n*** **= 5)**
LS BMD (g/cm^2^)	1.13 (1.05–1.23)	1.16	0.92 (0.84–0.99)	0.87	0.91 (0.82–1.01)	0.91 (0.71–0.96)	0.96 (0.86–1.07)	0.91 (0.79–1.04)
FN BMD (g/cm^2^)	0.89 (0.85–0.97)	1.00	0.73 (0.67–0.80)	0.80	0.69 (0.63–0.77)	0.63 (0.54–0.71)	0.76 (0.68–0.86)	0.71 (0.58–0.90)
TH BMD (g/cm^2^)	0.99 (0.91–1.06)	1.19	0.80 (0.74–0.88)	0.82	0.76 (0.70–0.85)	0.56 (0.56–0.71)	0.83 (0.74–0.92)	0.71 (0.56–1.00)
Calcium (mmol/L)	2.40 (2.32–2.46)	2.42	2.40 (2.34–2.48)	2.55	2.34 (2.26–2.43)	2.36 (2.15–2.43)	2.39 (2.31–2.46)	2.42 (2.26–2.50)
ALP (U/L)	165 (131–198)	164	158 (126–185)	140	154 (119–184)	170 (104–246)	158 (127–187)	164 (122–208)
Albumin (g/L)	44.0 (42.2–45.9)	46.2	43.8 (41.9–45.8)	45.4	42.2 (39.9–44.6)	42.6 (40.4–45.2)	43.5 (41.6–45.6)	45.2 (41.5–45.8)
***ADAMTS20* c.4090A>T**	**AA** **(** ***n*** **= 205)**	**AT** **(** ***n*** **= 13)**	**AA** **(** ***n*** **= 505)**	**AT** **(** ***n*** **= 29)**	**AA** **(** ***n*** **= 245)**	**AT** **(** ***n*** **= 15)**	**AA** **(** ***n*** **= 955)**	**AT** **(** ***n*** **= 57)**
LS BMD (g/cm^2^)	1.13 (1.06–1.23)	1.05 (1.04–1.18)	0.92 (0.84–0.99)	0.88 (0.83–1.02)	0.91 (0.82–1.00)	0.87 (0.74–0.99)	0.96 (0.86–1.07)	0.97 (0.84–1.05)
FN BMD (g/cm^2^)	0.89 (0.85–0.97)	0.88 (0.82–0.94)	0.74 (0.67–0.79)	0.74 (0.66–0.79)	0.69 (0.63–0.76)	0.73 (0.66–0.78)	0.76 (0.68–0.85)	0.76 (0.68–0.82)
TH BMD (g/cm^2^)	**1.00 (0.91–1.06)**	**0.91 (0.84–0.98)**	0.81 (0.74–0.88)	0.78 (0.73–0.87)	0.75 (0.69–0.85)	0.81 (0.73–0.83)	0.83 (0.74–0.92)	0.82 (0.75–0.90)
Calcium (mmol/L)	2.40 (2.32–2.47)	2.37 (2.29–2.41)	2.41 (2.34–2.48)	2.38 (2.34–2.43)	2.35 (2.26–2.43)	2.33 (2.27–2.37)	2.39 (2.31–2.46)	2.37 (2.33–2.41)
ALP (U/L)	165 (132–198)	167 (139-196)	159 (128–188)	152 (117–166)	154 (120–184)	155 (108-209)	158 (128–187)	155 (120–182)
Albumin (g/L)	44.0 (42.2–46.0)	43.8 (42.5–45.2)	43.7 (41.8–45.7)	44.6 (42.4–47.4)	42.3 (40.0–44.7)	41.9 (40.3–44.5)	43.5 (41.6–45.6)	43.8 (41.9–46.3)

^1^ Mann–Whitney U test computed only in controls with a normal BMD for the *ADAMTS20* c.4090A>T variant. ^2^ Women with an osteopenic and osteoporotic BMD at any measured sited without a fracture history. ^3^ Serum calcium reference range: 2.10–2.60 mmol/L. ^4^ Total serum ALP reference range: 100–290 U/L. ^5^ Serum albumin reference range: 35–55 g/dL. Significant associations highlighted in bold.

**Table 6:** Crude and Adjusted ORs with 95% CI for BMD and fracture risk. Logistic regression was computed for *ADAMTS20* c.4090A>T and *SELP* c.2177T>C variants. No analysis was conducted for *TGF-β2* c.1136C>T due to the low number of heterozygotes (*n* = 5).

**Genotype**	**Normal BMD *n* (%)**	**LS BMD *n* (%) ^1^**	**Crude OR (95% CI), *p***	**Adjusted OR (95% CI), *p* ^2^**
*SELP* c.2177T>C TT	331 (98.8)	4 (1.2)	1.0	1.0
TC/CC	434 (98.2)	8 (1.8)	1.53 (0.46–5.11), 0.494	1.55 (0.46–5.20), 0.482
*ADAMTS20* c.4090A>T AA	301 (93.8)	409 (94.9)	1.0	1.0
AT	20 (6.2)	22 (5.1)	0.81 (0.43–1.51), 0.507	0.77 (0.41–1.44), 0.415
**Genotype**	**Normal BMD *n* (%)**	**FN BMD *n* (%) ^3^**	**Crude OR (95% CI), *p***	**Adjusted OR (95% CI), *p***
*SELP* c.2177T>C				
TT	318 (98.1)	6 (1.9)	1.0	1.0
TC/CC	447 (98.7)	6 (1.3)	0.71 (0.23–2.23), 0.558	0.72 (0.23–2.27), 0.570
*ADAMTS20* c.4090A>T AA	293 (94.8	16 (5.2)	1.0	1.0
AT	417 (94.1)	26 (5.9)	1.14 (0.60–2.17), 0.685	1.06 (0.55–2.03), 0.854
**Genotype**	**Normal BMD *n* (%)**	**TH BMD *n* (%) ^4^**	**Crude OR (95% CI), *p***	**Adjusted OR (95% CI), *p***
*SELP* c.2177T>C TT	459 (98.3)	8 (1.7)	1.0	1.0
TC/CC	306 (98.7)	4 (1.3)	0.75 (0.22–2.51), 0.641	0.76 (0.22–2.57), 0.656
*ADAMTS20* c.4090A>T AA	426 (94.7)	24 (5.3)	1.0	1.0
AT	284 (94.0)	18 (6.0)	1.13 (0.60–2.11), 0.714	1.04 (0.55–1.97), 0.909
**Genotype**	**Controls *n* (%) ^5^**	**All-type Fractures *n* (%)**	**Crude OR (95% CI), *p***	**Adjusted OR (95% CI), *p* ^6^**
*SELP* c.2177T>C TT	765 (98.5)	12 (1.5)	1.0	1.0
TC/CC	263 (98.1)	5 (1.9)	1.21 (0.43–3.47), 0.720	1.16 (0.37–3.58), 0.800
*ADAMTS20* c.4090A>T AA AT	710 (94.4) 245 (94.2)	42 (5.6) 15 (5.8)	1.0 1.04 (0.56–1.90), 0.912	1.0 1.02 (0.54–1.94), 0.947
**Genotype**	**Controls *n* (%) ^5^**	**Wrist fractures *n* (%) ^7^**	**Crude OR (95% CI), *p***	**Adjusted OR (95% CI), *p* ^6^**
*ADAMTS20* c.4090A>T AA AT	710 (94.4) 107 (93.9)	42 (5.6) 7 (6.1)	1.0 1.11 (0.48–2.52), 0.811	1.0 1.12 (0.48–2.58), 0.799

^1^ Women with osteopenia and osteoporosis at the LS. ^2^ ORs adjusted for age. ^3^ Women with osteopenia and osteoporosis at the FN. ^4^ Women with osteopenia and osteoporosis at the TH. ^5^ All women without a fracture history irrespective of BMD. ^6^ ORs adjusted for age and BMD at the LS, FN and TH. ^7^ Other site-specific fractures (humerus, hip, vertebrae) could not be computed due to the absence or low number of heterozygous or homozygous alternative genotypes detected for the variants.

**Table 7:** Crude and adjusted ORs with 95% CI for *ADAMTS20* c.4090A>T in relation to serum calcium levels.

Calcium Tertile Intervals	*ADAMTS20* c.4090A>T		Crude OR (95% CI), *p*	Adjusted OR (95% CI), *p* ^1^
	AA	AT		
Highest	342 (97.2)	10 (2.8)	1.0	
Intermediate	285 (91.9)	25 (8.1)	3.00 (1.72–6.35), 0.004	2.99 (1.41–6.34), 0.004
Lowest	328 (93.7)	22 (6.3)	2.29 (1.07–4.92), <0.001	2.33 (1.08–5.03), 0.032

**^1^** ORs adjusted for age and intake of calcium supplements.

## Data Availability

All data presented in this study are not publicly available following the ethical guidelines. The data presented can be made available upon request from the corresponding author.
